# Third harmonic generation from the gold/amorphous silicon hybrid metasurface

**DOI:** 10.1515/nanoph-2021-0712

**Published:** 2022-04-20

**Authors:** Yang Li, Guanqing Zhang, Yutao Tang, Xuecai Zhang, Wenfeng Cai, Yanjun Liu, Tun Cao, Guixin Li

**Affiliations:** School of Biomedical Engineering, Dalian University of Technology, Dalian 116024, China; Department of Materials Science and Engineering, Southern University of Science and Technology, Shenzhen 518055, China; Department of Electrical and Electronic Engineering, Southern University of Science and Technology, Shenzhen 518055, China

**Keywords:** metasurface, plasmonics, third harmonic generation

## Abstract

The nonlinear optical properties of silicon have great potentials for developing all-optical switches and modulators, etc. Strategies based on all dielectric silicon photonic crystals and resonators have been proposed to design the nanophotonic devices with high nonlinearity. Nevertheless, the lack of compatible configuration with the mature CMOS technology may hinder the practical applications of the all dielectric devices. In this work, we proposed a metal–silicon hybrid metasurface to generate strong third harmonic signals from an amorphous silicon (α-Si) film. By integrating the α-Si film into a gold plasmonic nanocavity and controlling the periods of the gold meta-atoms, the efficiency of the THG process is expected to be greatly boosted. Compared to the planar α-Si film, the THG signal is enhanced by a factor of ∼370. The presented results in this work may open new routes for developing various silicon photonic devices with high optical nonlinearity.

## 1 Introduction

The silicon on insulator platform has become the research foundation of integrated photonics due to the high refractive index and low loss of silicon in the near-infrared frequency regime. In the areas of optical communications, interconnection, data storage, optical computing, silicon photonic chip plays important roles because of its capability of integrating electrons and photons on one chip [1, 2]. In the past two decades, the silicon on insulator platform has made many great achievements, including high-speed optical signal processing [3], [4], [5], [6], signal amplification and regeneration using four wave mixing process [7, 8], Raman lasing [9, 10], and wavelength conversion [11]. To meet the demand of on-chip light source with controllable wavelength output, nonlinear silicon photonic devices have been widely explored.

The pursuit of high-performance platform for nonlinear silicon photonics naturally aims at the intrinsic property of silicon. It is known that the central symmetry of silicon crystal inhibits its second-order nonlinear susceptibility *χ*^(2)^. By depositing a stressing silicon nitride thin film on top of a silicon waveguide, the strain induced *χ*^(2)^ can be realized [12]. Alternatively, by applying an external electric field to the silicon in a compact p-i-n junction, the third-order susceptibility *χ*^(3)^ of silicon can be used for electric field induced second harmonic generation (SHG) [13]. Moreover, SHG in silicon can be achieved by designing asymmetric silicon metasurface [14] or introducing symmetry breaking at materials’ surface [15], [16], [17]. However, the efficiencies of the SH waves in most of the silicon photonic devices are very low and thus limits the practical applications. In comparison, the *χ*^(3)^ of silicon does not suffer from the limitations of crystal symmetry. Therefore, the THG and four wave mixing processes in silicon photonics can be used for developing nonlinear optical sources.

THG can be observed in both bulk and amorphous silicon materials for pumping wave at near infrared wavelengths [18], however, the THG efficiency is not high due to the lack of mechanisms for light confinement and phase matching. To solve this problem, silicon photonic crystals, which consist of periodic unit cells with feature size of wavelength scale, were proposed to strongly localize the fields of fundamental waves to improve the THG efficiency [6, 19], [20], [21]. More recently, various resonant mechanisms, such as magnetic resonance [22], Mie resonance [23], Fano resonance [24, 25], and bound state in the continuum [26, 27] have been proposed to enhance the third-order nonlinear optical response of the silicon metasurfaces. On the other hand, plasmonic metasurfaces, which have excellent light localization properties, have been widely used for frequency conversions [28, 29]. By combining the plasmonic metasurfaces with semiconductor materials, the nonlinear optical efficiencies of the hybrid device could be further improved [30, 31].

In this work, we propose to enhance the THG efficiency of an amorphous silicon thin film by sandwiching it between the gold plasmonic meta-atoms and a gold mirror. The gold meta-atoms are the rectangular nanorods, the typical designs to enhance the nonlinear optical effects on the metasurfaces [28, 29]. With such rectangular meta-atoms, it is convenient to introduce localized plasmonic modes, which are sensitive to the polarization directions of the incident light. In addition, a perfect rectangular meta-atom has inversion symmetry, therefore the SHG process from the gold nanorod itself is negligible when the fundamental wave (FW) is normally incident [29]. The gold meta-atom, the silicon layer and a gold mirror form a nanocavity. The interplay of the localized plasmonic resonance of the gold meta-atoms and the gold–silicon–gold nanocavity effect are leveraged to boost the interaction of the fundamental wave (FW) with the tens of nanometer thick α-Si film. Compared with the planar α-Si film, the THG in the hybrid metasurface can be enhanced by ∼370 times.

## 2 Design, fabrication and characterization of the nanocavity metasurface

**Figure 1:**
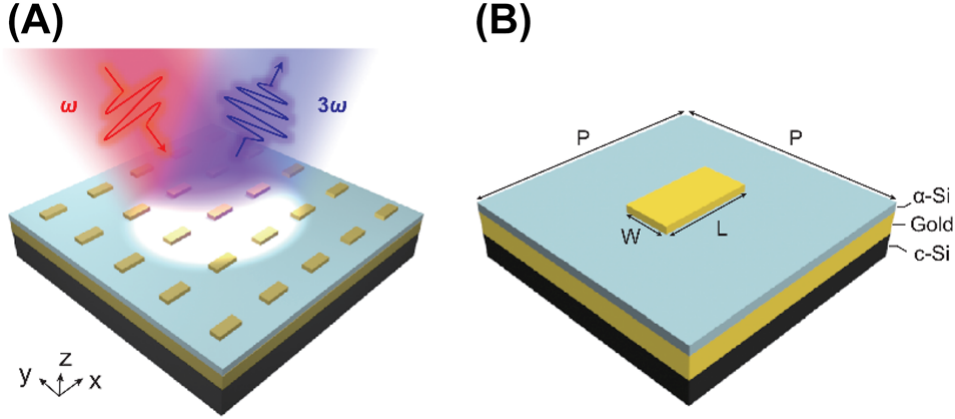
Schematic illustration of the THG from the gold/α-Si hybrid metasurface. (A) The metasurface consists of a layer of gold meta-atoms, an amorphous silicon spacer layer, and a gold mirror. Under the pumping of normally incident fundamental wave, the hybrid metasurface can radiate THG signals. (B) Geometrical parameters of a meta-atom, *P* is the period of the square lattice, *L* and *W* are the length and width of the gold meta-atom, respectively.

Figure 1A is the schematic illustration of the gold/α-Si hybrid metasurface. For a normally incident linearly polarized FW with frequency *ω*, the THG wave at the frequency of 3*ω* is generated from the metasurface. Figure 1B illustrates a unit cell of the hybrid metasurface. The rectangular gold meta-atoms are arranged in a square lattice with various periods from 400 to 900 nm along both *x*- and *y*-axes. The length (*L*) and width (*W*) of the gold meta-atoms are optimized to be 168 and 80 nm, respectively. The thickness of the gold meta-atoms, α-Si spacer, and gold mirror are set to be 30 nm, 83 nm, and 120 nm, respectively. The optimization of the structural parameters is to achieve a strong plasmonic resonance in the near infrared regime.

**Figure 2:**
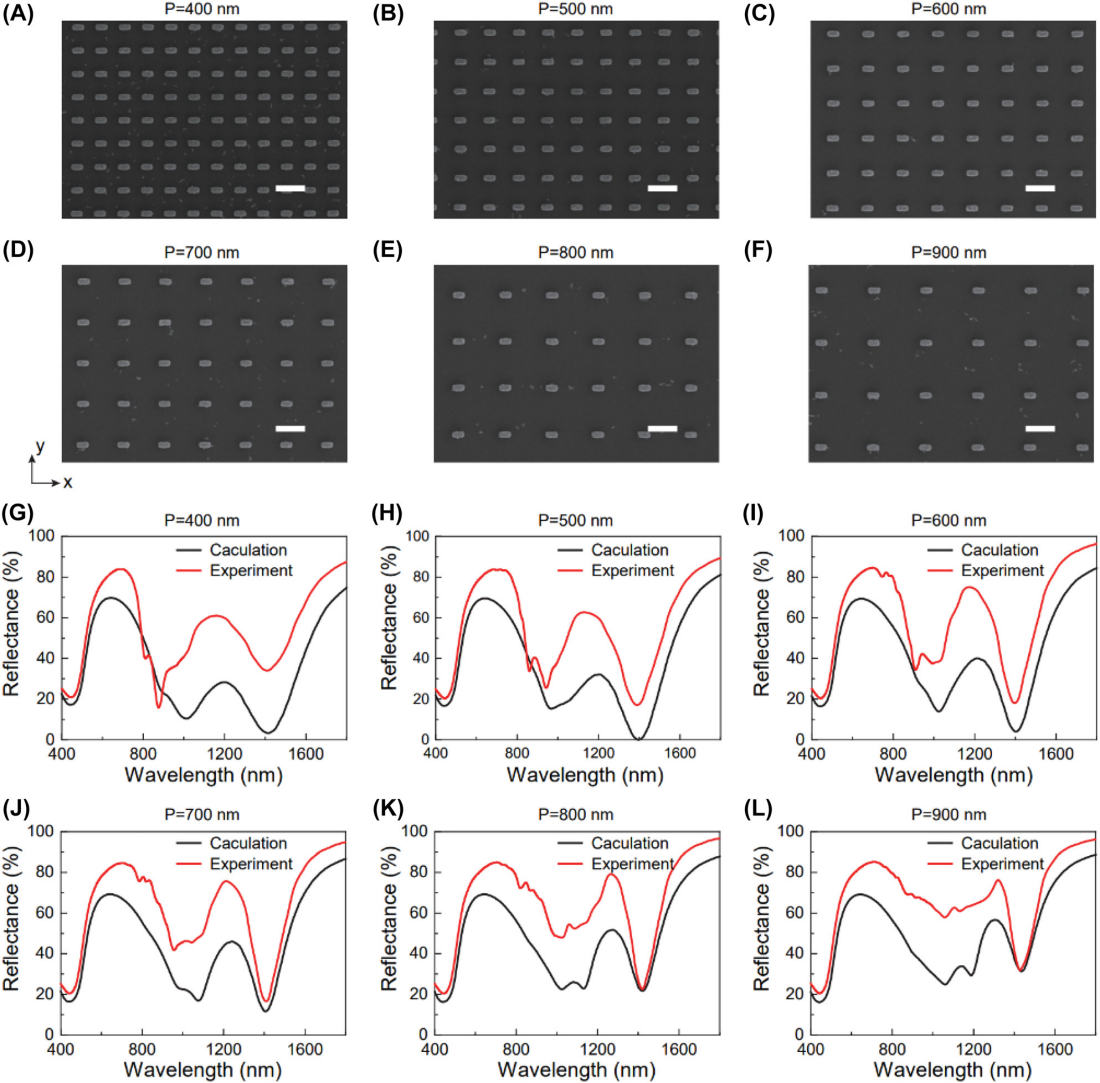
Linear optical properties of the gold/α-Si hybrid metasurfaces. Scanning electron microscope images of the metasurfaces with varying periods. (A) *P* = 400 nm, (B) *P* = 500 nm, (C) *P* = 600 nm, (D) *P* = 700 nm, (E) *P* = 800 nm, and (F) *P* = 900 nm. Scale bar: 500 nm. (G)–(L) Calculated and measured reflection spectra of the hybrid metasurfaces with different periods. The incident light is H-polarized (horizontally, along *x*-axis).

First, the nanofabrication of the gold/α-Si hybrid metasurfaces with various periods from 400 to 900 nm was conducted by using the electron beam lithography and the gold lift-off process. The hybrid metasurfaces were fabricated on a silicon substrate (Supplementary Materials S1). Figure 2A–F shows the scanning electron microscopy (SEM) images of the metasurfaces consisting of gold meta-atoms which are arranged in a square lattice. To know the linear optical properties of the metasurfaces with different periods, we measured their reflection spectra by using the microspectrophotometer (CRAIC 20/30 PV). It can be found that the resonant modes appear at the wavelengths between 1389 and 1430 nm. By using the measured complex refractive index of the α-Si thin film (Figure S1A), the reflection spectra of the six metasurfaces were also numerically calculated for incident light with linear polarizations along *x* (H-Pol) and *y* (V-Pol) directions. As shown in Figures 2G–L, the measured spectra (red lines) of the H-polarized light are in good consistency with the calculated ones (black lines). The deviations observed between the experimental and calculated results may arise from the imperfection of the fabricated metasurfaces.

By switching the polarization of the incident light from H to V direction, the resonant modes around 1400 nm disappear (Supplementary Figure S2). This means that the resonant modes come from the interplay between the localized plasmonic resonances along the long arm direction of the gold meta-atom and the cavity resonance. In comparison, there are no characteristic resonant modes from the gold/α-Si planar film. For wavelengths shorter than 700 nm, the reflection efficiency of the planar film device is very low, which is mainly due to the absorption of the α-Si thin film in the visible spectral region.

## 3 Nonlinear optical measurements

**Figure 3:**
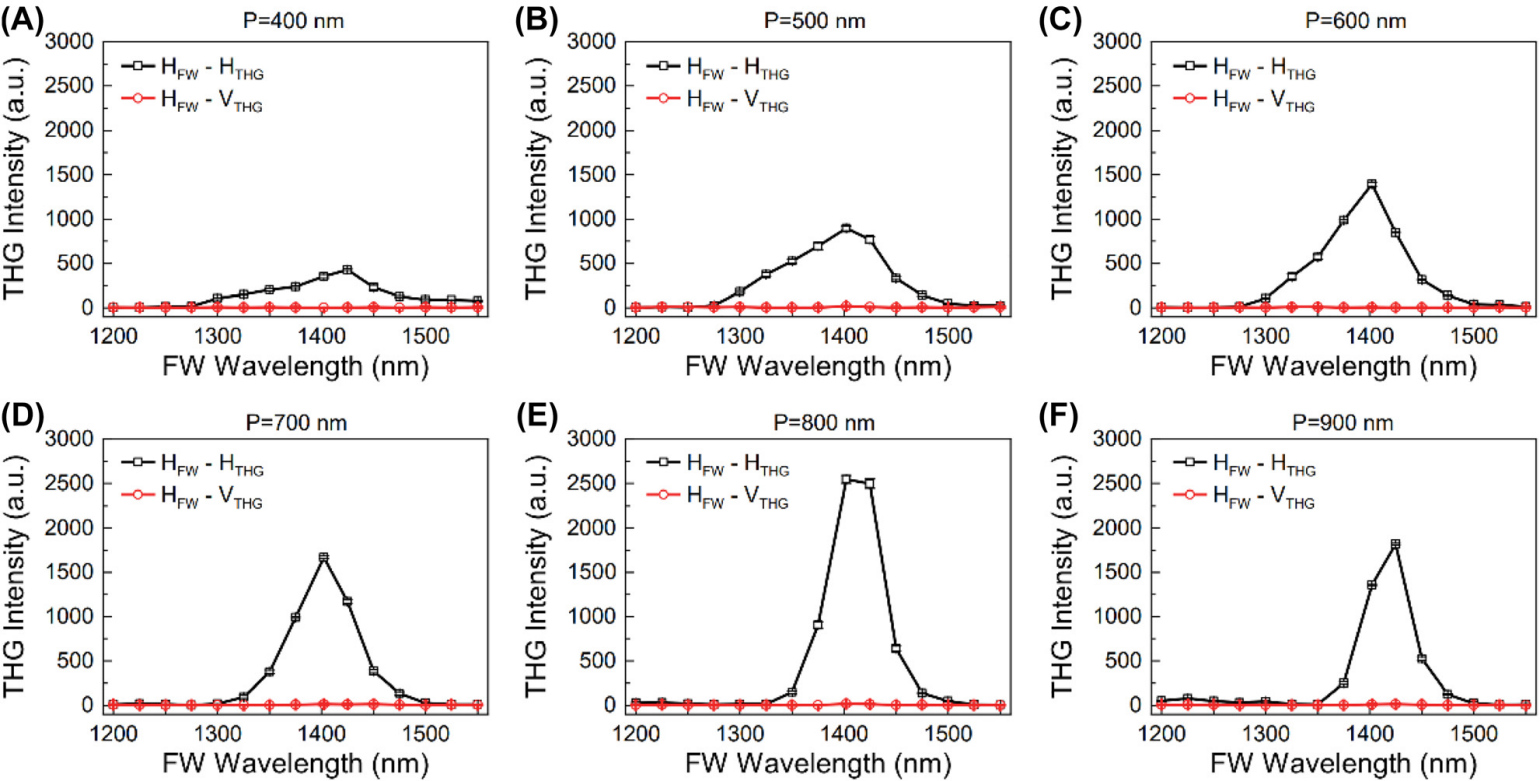
Measured wavelength dependent THG responses of the gold/α-Si hybrid metasurfaces with different periods. ‘H_FW_-H_THG_’ and ‘H_FW_-V_THG_’ represent the polarization states of the FW and the measured THG waves (H: horizontal polarization; V: vertical polarization). (A) *P* = 400 nm, (B) *P* = 500 nm, (C) *P* = 600 nm, (D) *P* = 700 nm, (E) *P* = 800 nm and (F) *P* = 900 nm.

We further investigated the nonlinear optical responses of the hybrid metasurfaces and the gold/α-Si planar film by using a spectrally tunable femtosecond laser (repetition rate: 80 MHz, pulse duration: ∼250 fs). The linearly polarized FW along H- and V-directions were focused onto the metasurfaces by an objective lens with NA of 0.1 (Figure S3). The THG signals were collected by the same objective lens and filtered by a dichroic mirror and finally recorded with a high sensitive spectrometer. Under the pumping of the linearly polarized FW with wavelength ranging from 1200 to 1550 nm, the properties of the THG signals emitted from the metasurface were measured. Figure 3A–F represent the wavelength dependent THG responses of the six hybrid metasurfaces under the excitation of the H-polarized FWs. The incident FWs with different wavelengths have the same power of ∼1.6 mW. The polarization properties of the THG signals were also analyzed. It can be found that the H-polarized THG signals are much stronger than the V-polarized ones. When the period of the meta-atoms is increased from 400 to 900 nm with 100 nm as a step, the peak THG intensity reaches a maximum at *P* = 800 nm. For this device, the strongest H-polarized THG response was observed at the fundamental wavelength of *λ* = 1402 nm, which is close to its linear optical resonance at *λ* ∼ 1422 nm. Figure S4A shows the THG spectra with H- and V-polarization for the H- and V-polarized FWs at *λ* = 1402 nm. The measured conversion efficiency of the H-polarized THG signals is about 5.2 × 10^−11^ under the pumping density of ∼42.5 MW/cm^2^. In Figure S4B, we also measured the power of the H-polarized THG signal as a function of the pumping power. The measured slope value of the power dependent curve is 2.87, which is close to the theoretical value 3.0 of the THG process. As shown in Figure S5, the polarization dependent THG responses of the gold/α-Si planar film were also characterized. At the fundamental wavelength of 1402 nm, it can be found that the intensity of THG signal from the hybrid metasurface is ∼370 times higher compared with that from the gold/α-Si planar film. This enhancement factor can be further improved by optimizing the deposition conditions of the α-Si thin film and the geometrical parameters of the gold meta-atoms.

## 4 Nonlinear optical calculations

**Figure 4:**
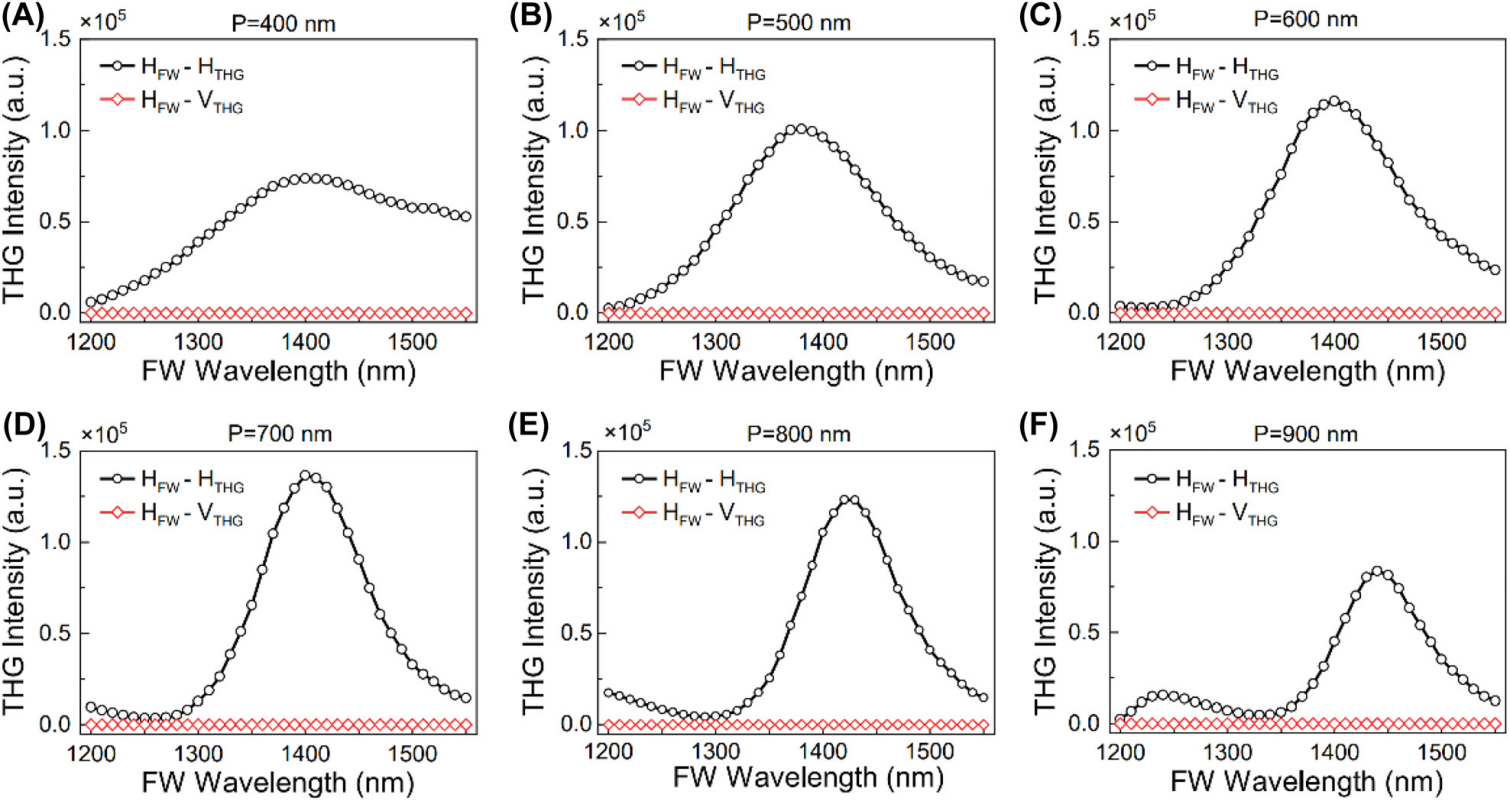
Calculated nonlinear optical responses of the gold/α-Si hybrid metasurfaces. Wavelength dependent H (parallel to *x*-axis) and V (parallel to *y* axis)-polarized THG components were calculated under the pumping of H-polarized FW. (A) *P* = 400 nm, (B) *P* = 500 nm, (C) *P* = 600 nm, (D) *P* = 700 nm, (E) *P* = 800 nm and (F) *P* = 900 nm.

**Figure 5:**
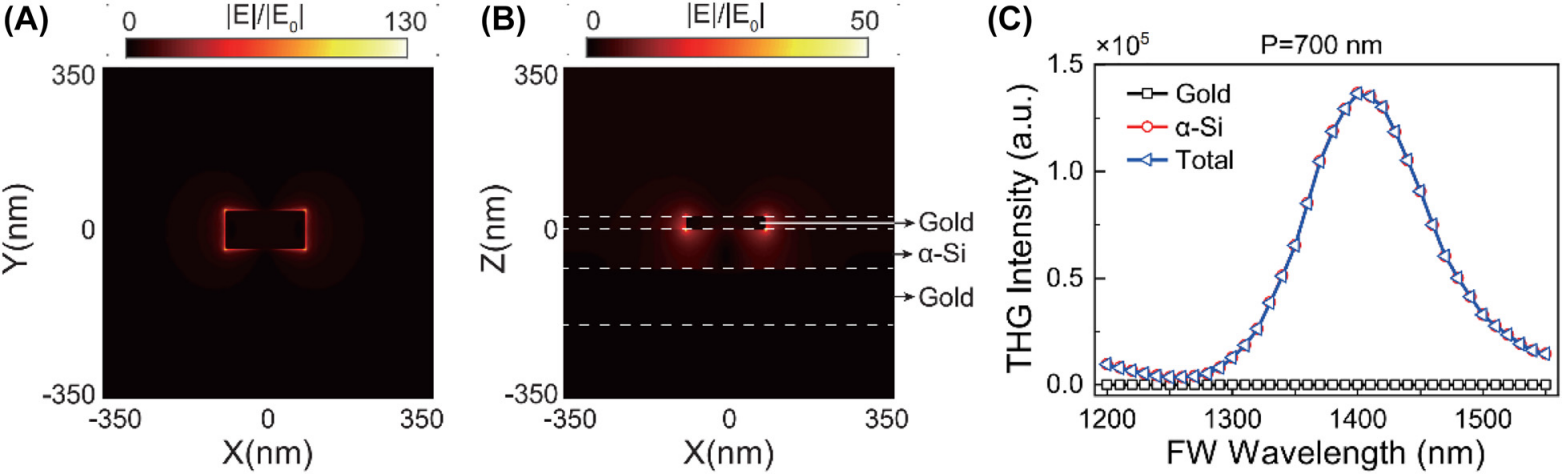
Calculated field distributions of the H-polarized FW and the THG contributions from gold and α-Si. (A) The calculated electric field distribution at the interface of gold meta-atom and the α-Si film. (B) The calculated electric field distribution in the *X*–*Z* plane (*y* = 0). The fundamental wavelength in (A) and (B) is *λ* = 1404 nm. (C) The calculated wavelength dependent THG intensities of the gold/α-Si hybrid metasurface.

In order to qualitatively predict the enhancement effects of the THG process from the gold/α-Si hybrid metasurfaces, we developed a COMSOL Multiphysics model to calculate the wavelength dependent THG responses [32]. The nonlinear polarization in the simulation is defined by $P_{3\omega }=\epsilon_{0}\chi^{\left(3\right)}E_{\omega }^{3}$ , where *E*_ω_ is the electric field at the fundamental frequency, *χ*^(3)^ is the third-order nonlinear susceptibility, and *ε*_0_ is the vacuum permittivity. In the COMSOL Multiphysics model, the gold meta-atom/α-Si acts as a polarized emitter to radiate the THG waves. The *χ*^(3)^ of gold and silicon used in the simulations are $\chi_{\text{gold}}^{\left(3\right)}=2\times 10^{-19}{\text{m}}^{2}{\text{/V}}^{2}$ and $\chi_{\alpha -\text{Si}}^{\left(3\right)}=2.79\times 10^{-18}{\text{m}}^{2}{\text{/V}}^{2}$ , respectively [33, 34]. As shown in Figure 4A–F, the wavelength dependent THG responses from the six metasurfaces were calculated for the FW with horizontal polarization. The peak THG responses are obtained at the fundamental wavelengths around 1380–1440 nm, which correspond to the resonant modes in the linear optical responses. The THG efficiency becomes higher when the period of the meta-atoms is increased and reaches the maximum for *P* = 700 nm. In comparison, the measured highest THG efficiency was observed from the metasurface with *P* = 800 nm. This deviation may come from the fact that the actual nonlinear susceptibilities of gold and silicon are slightly different from the ones in the literatures [33, 34]. To better understand the enhancement mechanisms of the THG process in the hybrid metasurface, the electric field distributions (*λ* = 1404 nm) at the interface of gold meta-atom/α-Si and in the *X*–*Z* plane (*y* = 0) were calculated for the metasurface with *P* = 700 nm. It can be found that the electric field is strongly localized at the interface of gold meta-atom/α-Si and inside the α-Si layer (Figure 5A and B). In Figure 5C, the wavelength dependent THG contributions from gold (black line with square symbol), α-Si (red line with circle symbol) and the whole hybrid metasurface (blue line with triangle symbol) are summarized. We can find that the THG signals from the silicon are much stronger than that of the gold meta-atoms, confirming the plasmon resonance mediated THG process in the silicon layer. Compared to the crystal silicon based dielectric metasurface [26], the THG efficiency of the gold/α-Si is still much lower. However, the hybrid configuration is more compact and has potential to be modulated by the external electric field [13].

## 5 Conclusions

In this work, we experimentally verified that the THG process of the gold/α-Si hybrid metasurface can be greatly enhanced through introducing the localized plasmon resonance and the design of a gold–silicon–gold nanocavity. Similar idea can be applied to the Kerr effect and four wave mixing process in the silicon based photonic devices. The hybrid metasurface architecture provides a promising route to develop nonlinear optical devices with desired optical functionalities. We expect that the concept and theoretical model proposed in this work may have important applications in developing ultracompact nonlinear optical sources, optical switches, high speed optical modulators and so on.

## Data availability

The data that support the findings of this study are available from the corresponding author upon reasonable request.

## Supplementary Material

Supplementary Material
