# Supplementary material for: Third harmonic generation from the gold/amorphous silicon hybrid metasurface
Source: Nanophotonics. 2022 Apr 20;11(10):2245–51. doi: 10.1515/nanoph-2021-0712 (PMC11635935; doi:10.1515/nanoph-2021-0712)
Supplement: Supplementary file 1 — Supplementary Material [file j_nanoph-2021-0712_suppl.docx]

Supplementary Information for

**Third harmonic generation from the gold/amorphous silicon hybrid metasurface**

Yang Li^1,2^, Guanqing Zhang^2^, Yutao Tang^2^, Xuecai Zhang^2^, Wenfeng Cai^3^, Yanjun Liu^3^, Tun Cao^1*^ and Guixin Li^2*^

^1^School of Biomedical Engineering, Dalian University of Technology, Dalian 116024, China

^2^Department of Materials Science and Engineering, Southern University of Science and Technology, Shenzhen 518055, China

^3^Department of Electrical and Electronic Engineering, Southern University of Science and Technology, Shenzhen 518055, China

*Email: caotun1806@dlut.edu.cn, ligx@sustech.edu.cn.

SI-1. Fabrication of the hybrid metasurface

Firstly, a 3 nm thick Cr adhesive layer, a 120 nm thick gold film and a 81 nm thick α-Si film were subsequently deposited onto the cleaned silicon substrate by using the electron beam evaporator (AUTO 500). Before vacuum deposition, the gold and α-Si target materials were pre-melted for 8 mins and 1 min, respectively. The deposition rate for the gold and α-Si thin films is about 0.89 Å/s and 0.54 Å/s, respectively. Then, a 129 nm thick electron resist (polymethyl methacrylate, PMMA) was spin-coated onto the gold/α-Si planar substrate. After that, the designed pattern was written into the PMMA layer by the electron-beam lithography, and then developed in the isopropanol diluted methyl isobutyl ketone (MIBK: IPA = 1:3). Subsequently, a 30 nm thick gold film was deposited by using the electron beam evaporator. At last, the gold/α-Si hybrid metasurface was obtained after the gold lift-off process.

SI-2. Linear optical measurement

The linear reflection spectra of the gold/α-Si hybrid metasurface and the planar film were measured by using the Microspectrophotometer (CRAIC 20/30 PV). Firstly, the linearly (horizontally and vertically) polarized incident light was focused on a 150 nm thick gold reference film, which was deposited on the silicon substrate, through a 10 × objective lens. The reflected light with parallel polarizations (H-H or V-V) were collected by using the same optical system and used as the reference signal. The polarization resolved reflection spectra (H-H, H-V, V-V and V-H) of the metasurfaces were then measured by the same optical system and were corrected after considering the reflectance of the reference device.

SI-3. Linear optical properties

Figure S1A shows the measured complex refractive index of the α-Si thin film. Figure S1B shows the calculated (blue line) and measured (red line) reflection spectra of the gold/α-Si planar film.


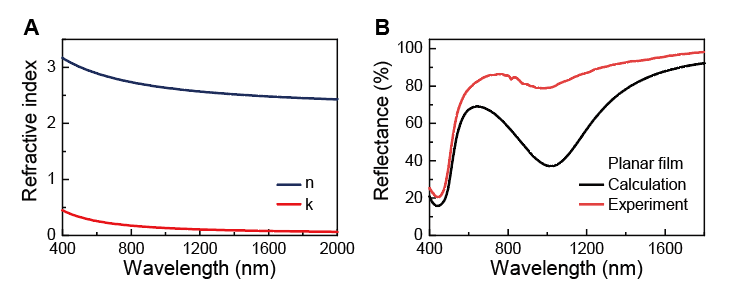


**Figure S1**: Linear optical property of the gold/α-Si planar film. (A) The measured complex refractive index of the α-Si thin film. (B) The calculated and measured reflection spectra of the gold/α-Si planar film.

As shown in Figures S2A-S2F, we also calculated and measured the linear optical responses of the metasurfaces for incident light with linear polarization along the y-axis (V-polarization). It can be found that the measured spectra (red lines) are in good consistence with the calculated ones (black lines). The deviations observed between the experimental and calculated ones may arise from the imperfections of the fabricated metasurafces.


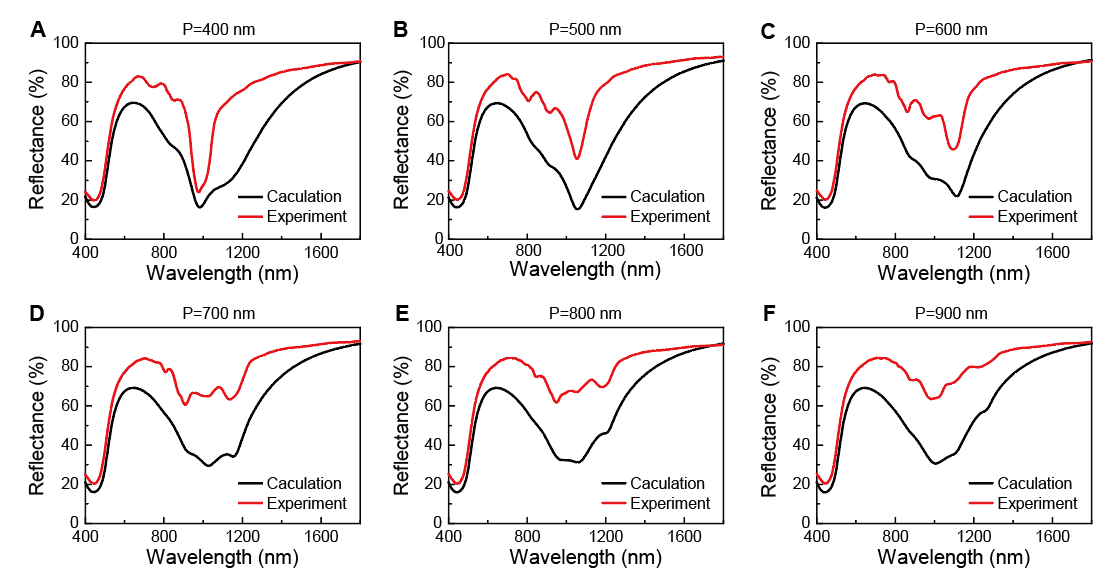


**Figure S2**: Linear optical properties of the gold/α-Si hybrid metasurfaces for V-polarized incident light. The calculated and measured reflection spectra of the hybrid metasurfaces with different periods. (A) 400 nm, (B) 500 nm, (C) 600 nm, (D) 700 nm, (E) 800 nm, (F) 900 nm.

SI-4. Nonlinear optical measurements

As shown in Figure S3, we characterized the THG responses of the gold/α-Si hybrid metasurface and the planar film by using a homemade optical setup. In this optical setup, an optical parametric oscillator (OPO) is pumped by a Ti: sapphire femtosecond laser (wavelength: 820 nm, repetition rate: 80 MHz). By adjusting the half-wave plate (HWP) and the linear polarizer (LP), we can control the intensity and polarization state of the FW. The incident near-infrared FW was focused onto the hybrid metasurface after passing through a 4 × objective lens. The THG signals were collected by an Andor spectrometer (SP500i) equipped with an EMCCD detector.


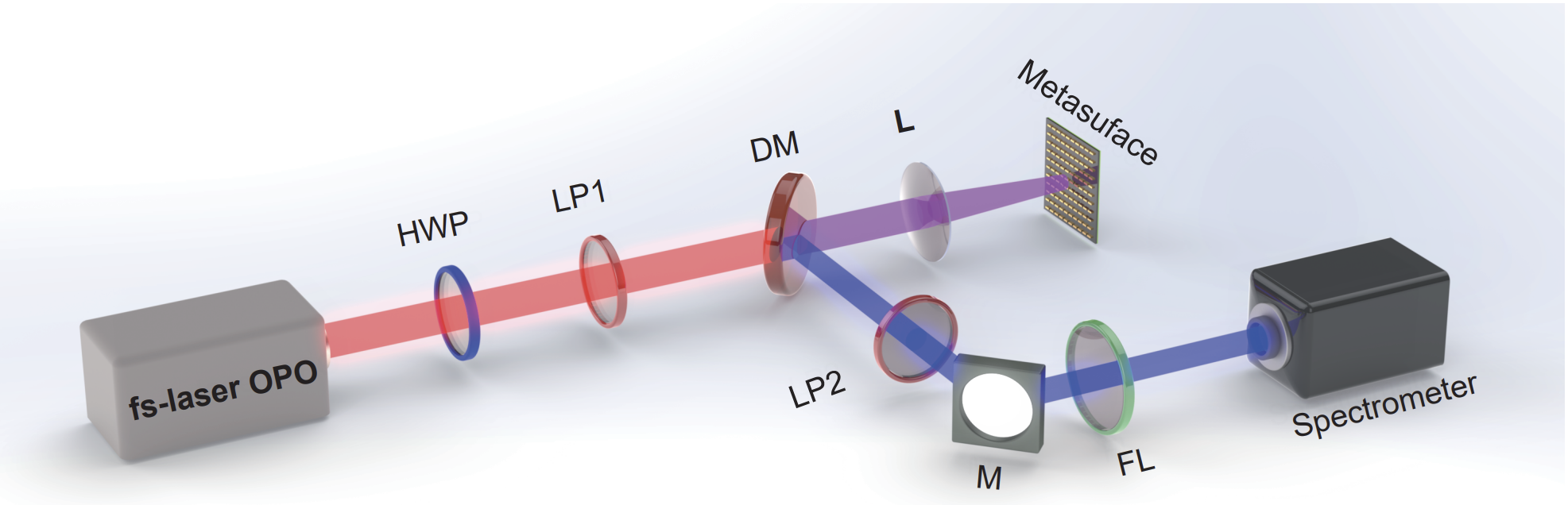


**Figure S3:** Optical setup for the measurement of the THG signals of the samples. fs-laser OPO: an optical parametric oscillator pumped by a femtosecond laser (Pulse duration: 250 fs, repetition rate: 80 MHz, central wavelengths: 1200 nm to 1550 nm), HWP: half-wave plate; LP1, LP2: linear polarizer; L: 4 × objective lens (Olympus, NA = 0.1); M: mirror; DM: dichroic mirror (Thorlabs, DMLP950); FL: short pass filter (Thorlabs, FESH0750); Spectrometer (Andor, SP500i).

SI-5. Nonlinear optical properties

Figure S4A shows the THG spectra with H- and V- polarization under the pumping of H- and V-polarized FW at λ=1402 nm. The incident power is ~ 1.6 mW. In Figure S4B, we measured the power of the H-polarized THG signals as a function of the H-polarized FW pumping power. The measured slope value of the power dependent curve is 2.87, which is close to the theoretical value of the THG process.


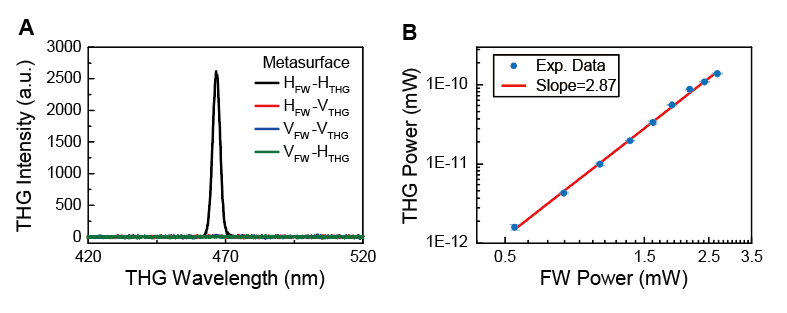


**Figure S4**: Measured THG responses of the gold/α-Si hybrid metasurface. (A) The measured H- and V- polarized THG signals from the hybrid metasurface for the H-polarized FW at λ=1402 nm. (B) The THG power is a function of the pumping power. The circle dots represent the experimental data, and the fitted slope value is 2.87.

As shown in Figure S5, we also measured the wavelength dependent THG responses with H- and V- polarization for the planar film under the pumping of the H-and V-polarized FWs, respectively. The incident FWs with different wavelengths have the same power of ~ 1.6 mW.


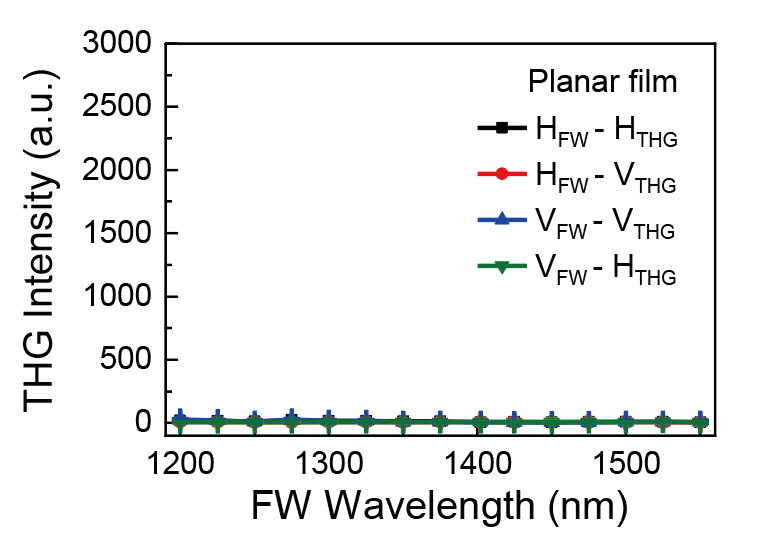


**Figure S5**: Measured nonlinear property of the gold/α-Si planar film. The measured wavelength dependent THG responses of the planar film. ‘H_FW_-H_THG_’, ‘H_FW_-V_THG_’, ‘V_FW_-V_THG_’ and ‘V_FW_-H_THG_’ represent the polarization states of the FW and the measured THG waves (H: horizontal polarization; V: vertical polarization).

SI-6. Nonlinear optical calculations

We calculated the nonlinear optical responses of the metasurface with P=700 nm under the pumping of V-polarized FW. Figures S6A and S6B show the calculated electric field distributions (λ=1404 nm) at the interface of gold meta-atom/α-Si and in the X-Z plane (y=0). Figure S6C shows the calculated THG intensities for the metasurface pumped by the V-polarized FW.


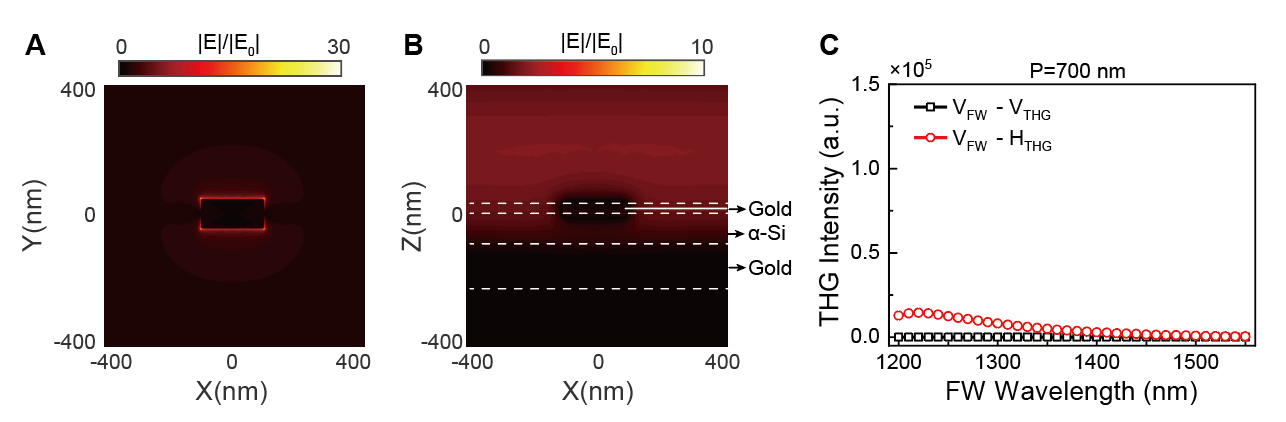


**Figure S6**: The calculated field distributions and the THG responses of the V-polarized FW. (A) The calculated electric field distribution at the interface of gold meta-atom and the α-Si film. (B) The calculated electric field distribution in the X-Z plane (y=0). The fundamental wavelength in (A) and (B) is λ= 1404 nm. (C) The calculated wavelength dependent THG intensities of the gold/α-Si hybrid metasurface.
